# Citrullinated Histone H3 Mediates Sepsis-Induced Lung Injury Through Activating Caspase-1 Dependent Inflammasome Pathway

**DOI:** 10.3389/fimmu.2021.761345

**Published:** 2021-12-07

**Authors:** Yuzi Tian, Patrick Li, Zhenyu Wu, Qiufang Deng, Baihong Pan, Kathleen A. Stringer, Hasan B. Alam, Theodore J. Standiford, Yongqing Li

**Affiliations:** ^1^ Department of Rheumatology and Immunology, Xiangya Hospital, Central South University, Changsha, China; ^2^ Department of Surgery, University of Michigan Health System, Ann Arbor, MI, United States; ^3^ National Clinical Research Center for Geriatric Disorders, Xiangya Hospital, Central South University, Changsha, China; ^4^ Provincial Clinical Research Center for Rheumatic and Immunologic Diseases, Xiangya Hospital, Central South University, Changsha, China; ^5^ Department of Internal Medicine, New York University (NYU) Langone Health, New York, NY, United States; ^6^ Department of Infectious Disease, Second Xiangya Hospital, Central South University, Changsha, China; ^7^ Department of Clinical Pharmacy, College of Pharmacy, University of Michigan, Ann Arbor, MI, United States; ^8^ Division of Pulmonary and Critical Care Medicine, University of Michigan Medical Center, Ann Arbor, MI, United States; ^9^ Department of Surgery, Feinberg School of Medicine, Northwestern University, Chicago, IL, United States

**Keywords:** citrullinated histone H3 (CitH3), sepsis, acute lung injury, Caspase-1 (CASP1), inflammasome

## Abstract

Sepsis is a life-threatening organ dysfunction caused by dysregulated host response to infection that often results in acute lung injury (ALI)/acute respiratory distress syndrome (ARDS). An emerging mechanism of sepsis-induced ARDS involves neutrophils/macrophages undergoing cell death, releasing nuclear histones to cause tissue damage that exacerbates pulmonary injury. While published studies focus on unmodified histones, little is known about the role of citrullinated histone H3 (CitH3) in the pathogenesis of sepsis and ALI. In this study, we found that levels of CitH3 were elevated in the patients with sepsis-induced ARDS and correlated to PaO2/FiO2 in septic patients. Systematic administration of CitH3 peptide in mice provoked Caspase-1 activation in the lung tissue and caused ALI. Neutralization of CitH3 with monoclonal antibody improved survival and attenuated ALI in a mouse sepsis model. Furthermore, we demonstrated that CitH3 induces ALI through activating Caspase-1 dependent inflammasome in bone marrow derived macrophages and bone marrow derived dendritic cells. Our study suggests that CitH3 is an important mediator of inflammation and mortality during sepsis-induced ALI.

## Introduction

Sepsis is a serious clinical problem with high morbidity and mortality (1–3). The ultimate cause of death in sepsis patients is multiple organ dysfunction. Acute lung injury (ALI), which is clinically manifest as acute respiratory distress syndrome (ARDS), represents the major devastating complication of sepsis (2, 4–7). Despite progress in the understanding of the pathophysiology of sepsis and organ dysfunction, the treatment relies largely on supportive care (8, 9). Therefore, researches to define the pathogenic mechanisms in sepsis and sepsis-induced ARDS are urgently needed to identify novel therapeutic targets.

One of the main pathophysiological events in of sepsis-induced ARDS is uncontrolled inflammation induced by cytokines and other inflammatory mediators. For example, host-derived danger-associated molecular patterns (DAMPs) like kidney mitochondrial, particularly cell-free mtDNA, developed during ALI/ARDS could act as a critical activator of the innate immune system and inflammation (10–12). Activation of the inflammasome pathway is one of the innate immune defenses triggered during ALI/ARDS (13). Upon sign of cellular ‘danger’, the signaling platform sensor protein recruits adaptor protein and effector protein to constitute a functional inflammasome. Generally, NLR Family Pyrin Domain Containing 3 (NLRP3) and Absent in Melanoma 2 (AIM2) are the most well-studied inflammasomes in ALI, with both pathways leading to the activation of effector protein Caspase-1 and the maturation of pro-inflammatory cytokines interleukin (IL)-1β and IL-18 (14–16). Elevated IL-1β and IL-18 play prominent roles in promoting inflammation in the lung. For example, elevated levels of circulating IL-18 are associated with a poor long-term prognosis in patients with sepsis-induced ARDS (17). In several different rodent models, IL-18/IL-1β neutralization or IL-1R signaling antagonism reduced lung injury (17–21).

A variety of danger signals are capable of activating inflammasomes. These include pathogen-associated molecular patterns (PAMPs) (22) and DAMPs, such as histone, adenosine triphosphate (ATP) and uric-acid crystals. Histones are important structural elements of the nucleosomes, which regulate gene expression and facilitate the formation of dense chromatin compaction (23). However, circulating histones have been found to be potent inflammasome activators (24). During sterile inflammatory liver injury, histones activate the NLRP3 inflammasome in Kupffer cells (25). In sepsis, extracellular histones mediate multiple organ dysfunction, such as cardiomyopathy (26) and ALI (27), through activating the NLRP3 inflammasome.

Citrullinated histone H3 (CitH3) is the post-translational modified form of histone H3. It is catalyzed by calcium-dependent enzymes peptidyl arginine deiminase 2 (PAD2) and peptidyl arginine deiminase 4 (PAD4). CitH3 has been recently found to be related to the severity of illness in patients with sepsis (28), and may represent a potential therapeutic target for endotoxic shock (29). However, the role of CitH3 in sepsis-induced ARDS is not yet clear. In this study, the expression of CitH3 in septic ARDS patients and septic ALI mouse model was determined. We then explored the effect of CitH3 peptide or neutralizing CitH3 administration in septic mouse model on lung injury and Caspase-1 activation in lung tissue. Finally, the direct effect of CitH3 on Caspase-1 activation in bone marrow derived macrophages (BMDMs) and bone marrow derived dendritic cells (BMDCs) *in vitro* was assessed.

## Materials and Methods

### Human Subjects

Three types of human samples were used in our study: 1) plasma from healthy controls and sepsis-induced ARDS patients, 2) bronchoalveolar lavage fluid (BALF) from healthy controls and sepsis-induced ARDS patients, 3) serum from sepsis patients who were then divided into sepsis patients with ARDS and sepsis patients without ARDS based on PaO2/FiO2 ratio.

Plasma and BALF samples were collected from sepsis-induced ARDS patients and healthy control subjects enrolled in the Acute Lung Injury Specialized Center of Clinically Oriented Research (SCCOR) as a part of a randomized trial of granulocyte-macrophage colony-stimulating factor administration (clinicaltrials.gov NCT00201409) conducted at the University of Michigan (30). Samples from only the placebo arm of the study were utilized. Healthy volunteers were asymptomatic, ambulatory non-smokers under 60 years of age, who had no known chronic medical conditions and were taking no medications. Serum samples and clinical data were collected from patients with sepsis during a consecutive enrollment observation cohort study conduct at the University of Michigan (28). Bronchoalveolar lavage fluid (BALF) was collected and processed by standard techniques. The sample preparation and patient information acquisition have been previously described (31). Human studies were approved by the University of Michigan Institutional Review Board (HUM00056630, IRB#2003-0430 and IRB#2003-0829). Written informed consents were obtained from all participants or their legal proxy for medical decision making before study inclusion.

### Mice

Experiments were performed using 8-15 weeks old C57B/6J male mice purchased from Jackson Laboratories. All animals were housed under specific pathogen-free conditions with free access to food and water. Animal studies were performed within the National Institutes of Health guidelines and were approved by the University of Michigan Animal Care and Use Committee (PRO00008861).

### CitH3 and H3 Peptides Synthesis

The CitH3 and H3 peptides were generated by New England Peptide Inc (Gardner, MA, USA). The sequence for H3 and CitH3 peptides are “[H2N-ARTKQTARKSTGGKAPRKQLATKAARKSAP-amide” and “[H2N-A(Cit)TKQTA(Cit)KSTGGKAP(Cit)KQLATKAA(Cit)KSAP-amide”, separately. The purity for both of them is ≥95% measured by HPLC. These peptides have been described in our recent publication (29, 32).

### Mouse Models of Acute Lung Injury

Acute lung injury in murine models were induced through two methods: 1) Cecum ligation and puncture (CLP) polymicrobial sepsis model or 2) administration of CitH3 peptide. Briefly, the peritoneal cavity was opened under inhaled isoflurane anesthesia. The cecum was exposed, ligated below the ileocecal valve using a 5-0 silk suture at 75% percent from the tip, and punctured through and through with a 21-gauge needle. For antibody neutralization experiments, our in-house developed CitH3 monoclonal antibody (26) with four citrulline residues (20mg/kg) or mouse immunoglobulin G (IgG) (20mg/kg) were intravenously injected 4 h after CLP. In the CitH3 peptide challenge model, the CitH3 peptide with four citrulline residues [A(Cit)TKQTA(Cit) KSTGGKAP(Cit) KQLATKAA(Cit)KSAP] (16.5 mg/kg) or vehicle was intravenously administered. For all the animal studies, mice were monitored for 10 days, or sacrificed at specific time points to harvest lung tissue, BALF and serum samples for mechanistic studies.

### Lung Injury Analysis

The harvested lung tissues were fixed in 4% paraformaldehyde, embedded in paraffin, and sliced into 5 μm sections. Hematoxylin and Eosin (H&E) staining was performed for histology detection. The ALI scoring was conducted by a pathologist blinded to the experiment groups. ALI was classified into 6 categories based on the parameters of 1) neutrophils, 2) septal hemorrhage and congestion, 3) septal mononuclear cell/lymphocyte infiltration, 4) alveolar hemorrhage, 5) alveolar macrophages, and 6) alveolar edema. The severity of each category was graded from 0 (minimal) to 3 (maximal) and the total score was calculated by adding the scores in each of these categories (28).

### BMDMs and BMDCs Isolation

BMDMs and BMDCs were isolated from WT mice using well-established protocols (33). In brief, bone marrow cells were flushed from the femur and tibia using 60 mL of Hank’s Balanced Salt Solution, and then incubated with specific completed medium. BMDMs were incubated with Iscove’s Modified Dulbecco’s Medium (IMDM) supplemented with 20% L929 cell culture medium, penicillin, streptomycin, 2-mercaptoethanol, glutamine, and 10% heat-inactivated fetal calf serum (FBS) (Gibco, Thermo Fisher Scientific). BMDCs were incubated with RPMI-1640 supplemented with 20 ng/ml rmGM-CSF (Biolegend #576302), penicillin, streptomycin, and 10% FBS. The medium was refreshed on day 3. Cells were harvested on day 7 for use in subsequent experiments.

### Cell Culture and Treatment

BMDMs and BMDCs were plated in a 12-well plate at the density of 106/ml in the above-mentioned complete medium. The next day, cells were treated with 30 µg/ml CitH3 (Cayman chemical, #17926) or H3 (Cayman chemical, #10263) protein dissolved in opti-MEM medium for 5 h. Cell-free supernatant was reserved for ELISA. Cell lysates were analyzed by Western blot with antibodies against Caspase-1 and β-actin.

### Western Blotting

Murine cell and tissue lysates were prepared with radioimmunoprecipitation assay (RIPA) buffer. Protein lysates were separated by sodium dodecyl-sulfate polyacrylamide gel electrophoresis (SDS-PAGE) and transferred to nitrocellulose membranes (Bio-Rad, Hercules, CA). Membranes were probed with anti-CitH3 (Abcam #ab5103, 1:1000 dilution), anti-Caspase-1 (Abcam # ab179515, 1:1000 dilution), anti-IL-1 β (R&D system, # AF-401-NA, 1:1000 dilution) and anti- β -actin (Cell Signaling Technology, #3700, 1:1000 dilution) antibodies, followed by HRP-conjugated anti-rabbit secondary antibody (Invitrogen, #G-21234) or Dylight 800-conjugated anti-mouse secondary antibody (Cell Signaling Technology, #5257S). Images were visualized with ChemiDoc™ Touch Imaging System (Bio-Rad) and analyzed with Image Lab (Bio-Rad).

### Enzyme-Linked Immunosorbent Assay (ELISA)

Concentrations of CitH3 were measured by an in-house developed ELISA as previously described (32). Levels of IL-1 β, IL-18 and TNF-α in cell supernatant and BALF were measured by the ELISA core in the University of Michigan using the core-developed sandwich ELISA (34, 35).

### Statistical Analyses

For the human data, the Pearson-D’Agostino normality test was first applied. Since none of the comparisons passed the normality test, the non-parametric Mann-Whitney U test was used to compare differences between the two groups.

For mouse *in vivo* and *in vitro* studies, parametric tests were used because the sample size was too small for normality testing. The unpaired two-sided t test was used to compare differences between two groups, and the one-way analysis of variances (ANOVA) followed by Bonferroni’s multiple comparisons test was used to compare multiple groups. The survival curve was analyzed by log-rank test.

A p-value less than 0.05 was considered significant for all experiments. Statistical analyses were conducted, and figures generated, using Prism software (GraphPad, San Diego, CA).

## Results

### CitH3 Is Increased in Septic ARDS Patients and CLP-Induced Animal Model of Sepsis

**Figure 1:**
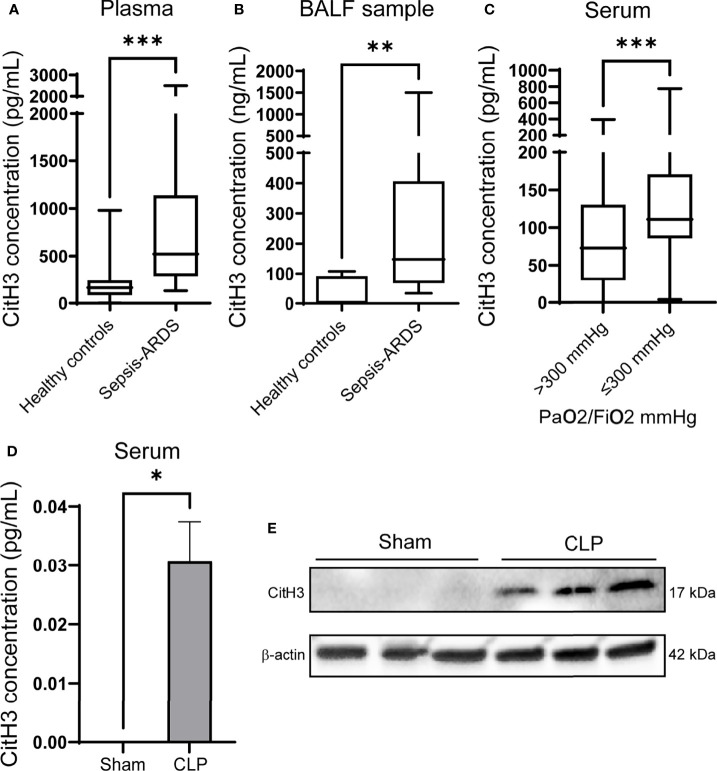
CitH3 is increased in septic ARDS patients and CLP-induced animal model of sepsis. Levels of CitH3 in **(A)** plasma and **(B)** BALF from healthy controls and patients with sepsis-induced ARDS (n = 19 *vs* 13 for plasma samples; n = 7 *vs* 11 for BALF samples). **(C)** Circulating CitH3 in septic patients with PaO2/Fi02 > 300 mmHg (n=90) and ≤300 mmHg (n = 44). **(D)** Levels of serum CitH3 in sham and CLP-induced sepsis-ALI mouse model at 12 hours (n = 3/group). **(E)** Western blot results show the expression of CitH3 in mouse lung tissue with or without CLP (n = 3/group). Nonnormality data are expressed as minimal to maximal value with quantile range **(A–C)**. Data in D are expressed as mean ± SEM. *P < 0.05, **P < 0.01, ***P <0.001. SEM, standard error of the mean.

Sepsis is a common predisposing factor for ARDS. We measured the levels of CitH3 in the plasma and BALF from patients with sepsis-induced ARDS. Compared with healthy volunteers, patients with sepsis-induced ARDS had significantly higher CitH3 concentrations within 7 days of ARDS onset. The elevations of CitH3 were found in both the circulation (**Figure 1A**) and in alveolar space (**Figure 1B**). At the same time, we divided septic patients into two groups based on the PaO2/FiO2 index, and septic patients with PaO2/FiO2 < 300 mmHg showed significantly higher levels of circulating CitH3 (**Figure 1C**). Previously, we have repeatedly showed that CLP induced ALI in the murine model (35, 36). To see the coherence between human sepsis-ARDS and murine sepsis-ALI, the levels of CitH3 were measured in mouse serum and BLAF. Consistent with the results of human ARDS patients, the concentrations of CitH3 were elevated in both serum (**Figure 1D**) and lung tissues (**Figure 1E**) of the CLP-induced ALI. Together, our results suggest that CitH3 may be involved in the development of ALI/ARDS.

### Systemic Administration of CitH3 Peptide in Mice Leads to Its Accumulation in Lung Tissues and Induces Expression of Endogenous CitH3 Protein

**Figure 2:**
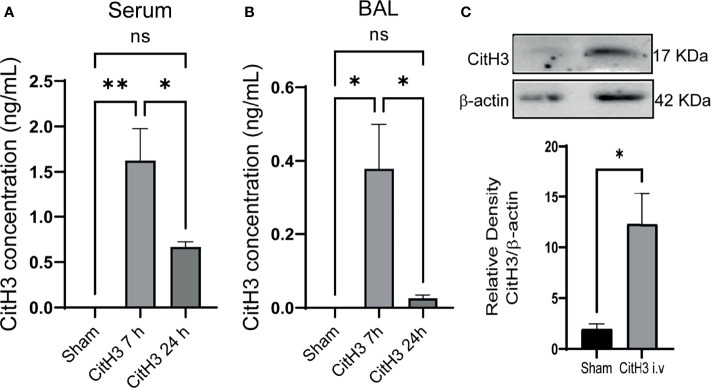
Intravenous injection of Cith3 in mouse induces its accumulation in lung tissue. Levels of CitH3 in **(A)** serum (n = 3, 4, 4 for each group) and **(B)** BALF (n = 3, 5, 5 for each group) in mouse after i.v. injection of CitH3 peptide at 0 h, 7 h and 24 h. **(C)** Representative Western blot images and densitometry quantification of expression of CitH3 protein in mouse lung tissue with or without CitH3 peptide challenging (n = 3/group) at 24 h. Data are expressed as mean ± SEM. *P < 0.05, **P < 0.01. i.v., intravenous; ns, not significant; SEM, standard error of the mean.

To determine the effect of the CitH3 on lung injury responses *in vivo*, mice were systemically challenged with CitH3 peptide through tail vein injection. The distribution of CitH3 peptide was examined by ELISA afterward. At baseline, the concentration of CitH3 was not detected (<20 pg/ml) in the serum. However, CitH3 was enriched at a high concentration in the serum at 7 h and decreased by 24 h (**Figure 2A**). A similar trend of CitH3 was also found in mouse BALF (**Figure 2B**). Since ELISA was not able to distinguish the signal of exogenous Cith3 peptide from endogenous CitH3 protein, Western blot was performed. We showed the level of CitH3 protein (17 kDa) was also increased in the lung tissue after CitH3 peptide (3.5 kDa) treatment (**Figure 2C**). The results indicate CitH3 peptide injection leads to accumulation and expression of CitH3 in lung tissues.

### Systemic Administration of CitH3 Peptide in Mice Induces Caspase-1 Activation and Lung Injury

**Figure 3:**
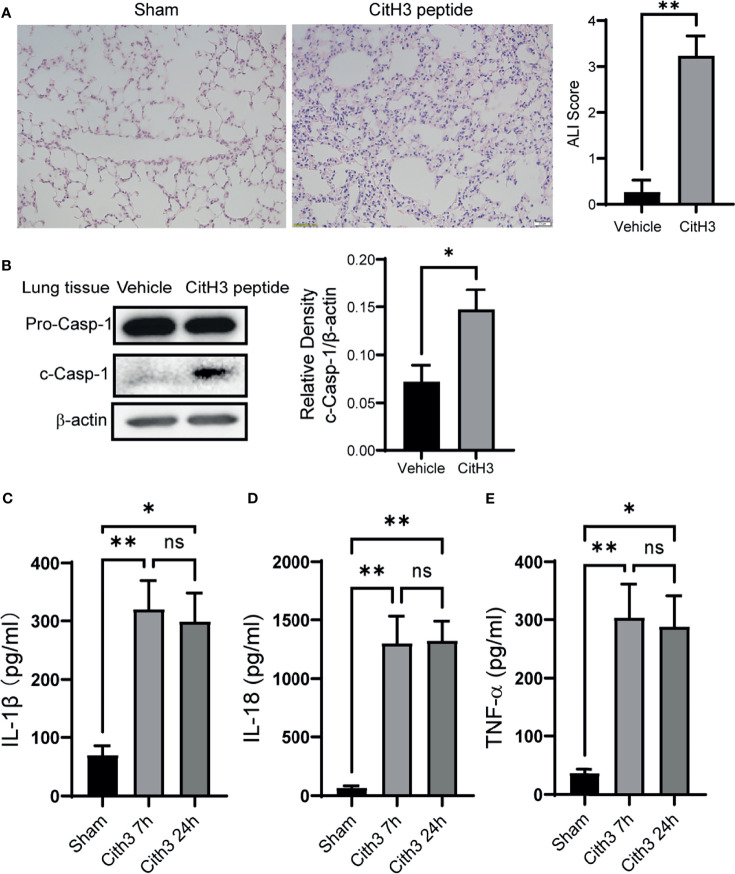
Intravenous injection of CitH3 induces Caspase-1 activation in lung tissue. **(A)** Representative histology images of murine lungs and ALI score following H&E staining in Sham and CitH3 treatment group. **(B)** Representative Western blot images and densitometry quantification of activation of Caspase-1 in mouse lung tissue with or without CitH3 peptide challenging (n = 3/group). Concentrations of **(C)** IL-1β, **(D)** IL-18, and **(E)** TNF-α in the BALF in mouse after i.v. injection of CitH3 peptide at 0 h, 7 h, and 24 h (n = 4/group). Data are expressed as mean ± SEM. *P < 0.05, **P < 0.01. i.v., intravenous; ns, not significant, SEM, standard error of the mean.

The histology of lung tissue was examined in mice with CitH3 peptide treatment. Compared with the vehicle group, mice treated with CitH3 showed pathological features of ALI, manifested as more inflammatory infiltration, alveolar hemorrhage, pulmonary congestion, edema, and thickening of the alveolar wall (**Figure 3A**). We hypothesized that extracellular CitH3 mediated ALI through activating Caspase-1 dependent inflammasome. Caspase-1 activation and cytokine release were examined. The activation of Caspase-1 in the lung tissue of the CitH3 treatment group was significantly increased (**Figure 3B**), and the release of IL-1β, IL-18 and TNF-α in BALF was also observed (**Figures 3C–E**).

### CitH3 Activates Caspase-1 Dependent Inflammasomes in BMDMs and BMDCs

**Figure 4:**
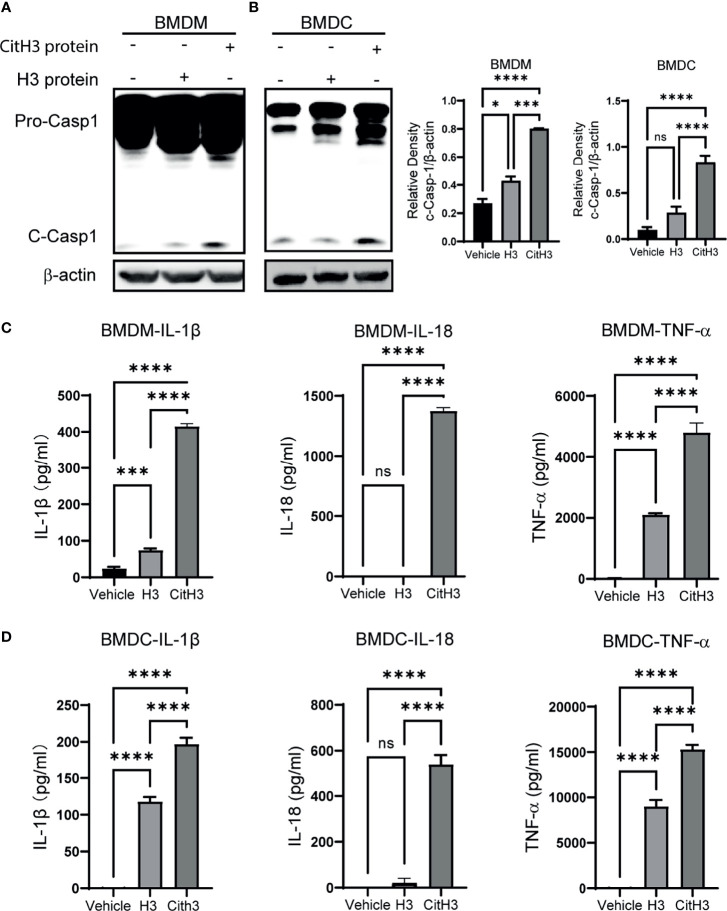
CitH3 induces activation of Caspase-1 in BMDMs and BMDCs. BMDMs and BMDCs were isolated from WT mice. Representative Western blot images and densitometry quantification of activation of Caspase-1 in **(A)** BMDMs (n = 3) and **(B)** BMDCs (n = 5) after treatment with vehicle, H3 or CitH3 for 5 h. Concentrations of IL-1β, IL-18 and TNF-α in the supernatant of **(C)** BMDMs (n = 4/group) and **(D)** BMDCs (n = 5/group) after treatment with vehicle, H3 or CitH3 for 5 h. Results are representative of more than 3 independent experiments. Data are expressed as mean ± SEM. *P < 0.05, ***P < 0.001, ****P < 0.0001. ns, not significant; SEM, standard error of the mean.

To further verify the activation effects of CitH3 protein on Caspase-1 dependent inflammasomes, we used CitH3 protein to stimulate BMDMs and BMDCs *in vitro*. The CitH3 protein induced robust Caspase-1 activation in both BMDMs and BMDCs, which was manifested by increased Caspase-1 cleavage (**Figures 4A, B**) and IL-1 β and IL-18 secretion (**Figures 4C, D**). It has been reported that histone H3 protein activates Caspase-1 dependent inflammasomes, such as NLRP3, and triggers sterile inflammation. In this study, CitH3 protein was more effective than H3 peptide in inducing the activation of Caspase-1 and elevation of IL-1β and IL-18. It is worth noting that TNF-α, an important transcription regulator of the inflammasome (37), was also significantly up-regulated after treatment with the CitH3 protein in BMDMs and BMDCs (**Figures 4C–D**).

### Neutralization of CitH3 Decreases Mortality and Lung Injury in Sepsis-Induced ALI Model

**Figure 5:**
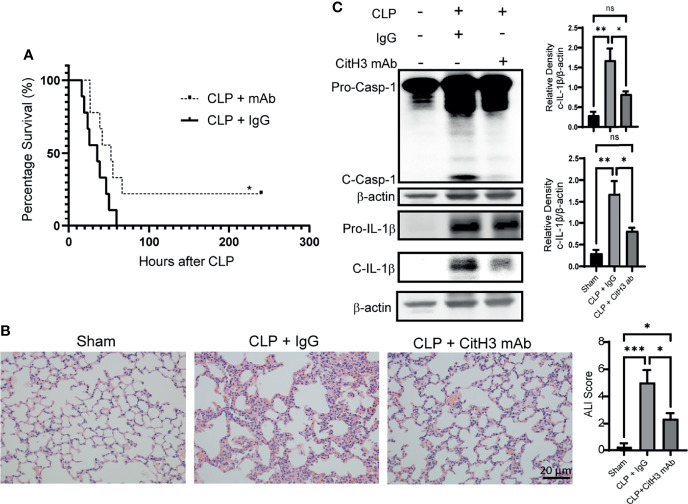
Neutralization of CitH3 in murine CLP model decreases mortality and Caspase-1 induced lung injury. WT mice were subject to CLP model and then intravenously (4h after CLP) treated with 20 mg/kg IgG or CitH3 monoclonal antibody. **(A)** The survival curve of mice with or without CitH3 monoclonal antibody in CLP model (n = 9/group). **(B)** Representative histology images of murine lungs and ALI score following H&E staining in Sham, CLP with IgG or CitH3 monoclonal antibody treatment group at 24 h. **(C)** Representative Western blot images and densitometry quantification of activation of Caspase-1 (n = 3/group) and cleavage of IL-1β (n = 4/group) in the lung tissues of Sham, CLP with IgG or CitH3 monoclonal antibody treatment group at 24 h. Data are expressed as mean ± SEM. *P < 0.05, **P < 0.01, ***P <0.001. ns, not significant; SEM, standard error of the mean.

We further showed that neutralizing CitH3 by intravenous injection of CitH3 antibody significantly increased the survival rate of CLP-induced septic mice, compared with the IgG group (**Figure 5A**). As expected, the lung injury was also attenuated in the CitH3 antibody treatment group (**Figure 5B**). CLP augmented significant Caspase-1 activation in the lung tissue, and treatment with CitH3 antibody attenuated this effect as assessed by pro-Caspase-1 and pro-IL-1β cleavage (**Figure 5C**).

## Discussion

In the current study, we have demonstrated for the first time that the expression of CitH3 was increased in the serum and BALF of the patients with sepsis-induced ARDS. In addition, the CitH3 levels in the septic patients with PaO2/FiO2 ≤ 300 were significantly higher than that of sepsis patients with PaO2/FiO2>300. Systematic administration of CitH3 peptide in mice provokes Caspase-1 activation in the lung tissue and causes acute lung injury, which may be mediated by CitH3-induced Caspase-1 activation in BMDMs and BMDCs. Neutralization of CitH3 with a monoclonal antibody in the CLP-induced sepsis model improves survival and attenuates acute lung injury as well as pro-Caspase-1 cleavage in the lung tissue.

Extracellular histone H3 has previously been identified as a potential mediator of sepsis and sepsis-induced organ injury (27, 38, 39). However, histone H3 could undergo various post-translational modifications (PTM) under stress before being released, such as phosphorylation, acetylation, methylation, ubiquitination and citrullination. These PTMs are recognized as important mechanisms for regulating gene expression, but few studies have focused on the effects of these modified histones on sepsis and ALI after they are released from the cell. CitH3 is a key component released from cells during the formation of neutrophil extracellular traps (NETs), which is a form of neutrophil death triggered by stimuli such as microbial, DAMPs and other noxious agents. In 2011, our team first identified CitH3, the post-translational form of H3, as a potential serum protein biomarker in a lethal model of lipopolysaccharides (LPS)-induced shock (40). Over the last decade, we have made significant progress in establishing CitH3 as a reliable biomarker for septic patients and the concentrations of CitH3 in blood correlated with disease severity (28). Moreover, we demonstrated chemical interventions that inhibit CitH3 are beneficial in mouse models of both endotoxic and septic shock. Targeting CitH3 is a potential therapeutic strategy for the mouse model of endotoxic shock (29). Consistent with our findings, Tsung et al. found that CitH3 was elevated in lung tissue of murine ALI model as part of component of NETs released during ALI (41). Besides, Yuan’s team found that CitH3 caused endothelial barrier dysfunction (42). And histone H3 citrullination has been reported to reduce antibacterial activity and exacerbates proteolytic degradation of histone H3 (43). The current study revealed an association between extracellular CitH3 and the severity of sepsis-ALI and possible mechanisms involved, identifying CitH3 as a potentially viable therapeutic target to reduce the severity and consequences of sepsis-ALI.

While this study focused on sepsis-ALI/ARDS, we found that the levels of CitH3 were elevated in both the alveolar space and in the circulation of septic patients, suggesting that CitH3 may not only play a role in local inflammatory response but also the systemic inflammatory changes seen in sepsis. However, after challenging mice with CitH3 peptide, the lung tissue showed the most significant elevation of endogenous CitH3 protein compared to other tissues (**Supplemental Figure 1**), which indicates that CitH3 may preferentially localize in the lung. That is in accordance with the fact that lung is a highly vulnerable organ in sepsis. It is tempting to speculate that circulating CitH3 peptide that accumulates in lung tissue after tail vein injection may directly or indirectly activate neutrophils or other immune cells, and ultimately lead to the passive or active release of CitH3 protein.

As with sepsis-ALI/ARDS, we suspect NETs are the important source of CitH3. Systematic inflammation and infection induce neutrophils to migrate to the lung tissue and release the NETs to sequester bacteria, which is a mechanism for pathogen inactivation proposed previously. NETs, which contain a complex of chromatin fibers mixed with granule-derived antimicrobial peptides and enzymes, trap and kill bacteria (44–46). While NETs can help to target and trap bacteria, the released CitH3 aggravates pulmonary inflammation in lung by activating Caspase-1 dependent inflammasomes in BMDMs and BMDCs, which may further exacerbate lung injury.

Caspase-1 mediated inflammasome activation in macrophages and dendritic cells plays an important role in ALI (47). In the present study, CitH3 induced Caspase-1 activation in both BMDMs and BMDCs. Of note, to activate Caspase-1 *in vitro*, it normally requires PAMPs like LPS priming process to induce production of pro-Caspase-1, pro-IL-1β and pro-IL-18. However, CitH3 treatment not only can induce Caspase-1 activation, but also could induce significant elevation of pro-Caspase-1 and pro-IL-1β itself (**Supplemental Figure 2**). We observed that incubation of BMDMs and BMDCs with CitH3 protein can also release large amounts of TNF-α in the supernatant. TNF-α was found to activate selectively the NLRP3 inflammasome without the requirement for a priming signal through the TNF receptor–Caspase-8–Caspase-1 pathway (48, 49). Besides, it was reported that TNF-α is an important transcriptional regulator of inflammasome components (50). Tnf–/– BMDCs and mice showed a marked reduction expression of inflammasome components, including pro-Caspase-1, pro-IL-1b and pro-IL-18 (37). Thus, we suspect that CitH3 protein could trigger both NF-Kβ and Caspase-1 pathway activation. The TNF-α released from BMDMs and BMDCs by CitH3 treatment could activate TNF receptor–Caspase-8–Caspase-1 pathway directly or induce the expression of pro-Caspase-1, pro-IL-1β and pro-IL-18. At the same time, CitH3 activates the inflammasome, leading to the cleavage of pro-Caspase-1, pro-IL-1β and pro-IL-18.

The lungs are populated by macrophages, which are equipped with a set of pattern recognition receptors (PRRs), including Toll-like receptors (TLR) and scavenger receptors, readily respond to DAMPs. Several studies have shown that inflammation caused by TLR activation by endogenous ligands participates in the development of ALI/ARDS (51, 52). In contrast to the homeostatic apoptosis, highly pro-inflammatory necrotic types of cell death, such as necroptosis and pyroptosis tend to trigger an inflammatory response during ALI/ARDS. In this current study, CitH3 peptide also induced cell death after treatment in BMDMs (**Supplemental Figure 3**). Therefore, in addition to releasing IL-1β and TNF-α, CitH3 may also exaggerate ALI by releasing DAMPs into the lung tissue through Caspase-1 mediated pyroptosis pathway. In contrast to the IL-1β release and Caspase-1 cleavage, the H3 treatment group showed comparable cell death to the CitH3 treatment group (**Supplemental Figure 3**). This indicates that CitH3 and/or H3 may activate multiple cell death pathways besides Caspase-1 mediated cell death. Actually, the high concentration of TNF-α released after treatment with CitH3 and H3 can also induced other lytic cell death like necroptosis (53). Interestingly, a recent study showed that prolonged exposure of myeloid cells to DAMPs like oxLDLs can induce cell to switch between cell death pathways (54). CitH3 and H3 may also activate different cell death pathways simultaneously or undergo a transition between different cell death pathways. Altogether, the DMAPs released by CitH3 induced cell death may also be an important effector mechanism of CitH3.

CitH3 is catalyzed by PAD2 and PAD4, two enzymes present in both neutrophils and macrophages. We used citrullinated histone H3 (R2/R8/R17/R26) peptide to develop the CitH3 mAb antibody used in this study. This CitH3 mAb recognizes epitopes on CitH3 that are specific for both PAD4 (R2/R8/R17) (55) and PAD2 (R26) (56). Therefore, it can more effectively sequester CitH3 generated by both PAD2 and PAD4 as compared with the commercial CitH3 antibody bound only to the epitope of R2/R8/R17 (29), which makes it a potentially effective agent for treating sepsis-ALI in animal models.

This study has several limitations. We only identified the role for CitH3 in a murine CLP model of sepsis-induced ALI, whereas its role in other ALI models (such as pneumonia-ALI) requires further study. In addition, our study showed that CitH3 mediates lung injury through Caspase-1 dependent inflammasome pathway. However, multiple inflammasomes such as NLRP3, AIM2, and pyrin, could lead to activation of Caspase-1. Both AIM2 and NLRP3 have been reported to participate in the pathogenesis of ALI/ARDS (57, 58). It is unclear whether CitH3 induced Caspase-1 cleavage due to the activation of NLRP3 or other inflammasomes, or a combination of multiple different ones. Moreover, our *in vitro* experiments demonstrated that CitH3 significantly induced more Caspase-1 activation compared to H3. Even so, it is still important to determine whether such effect could be extended to all *in vivo* studies. Further investigations are required to have a better understanding of the precise mechanisms in the future.

In conclusion, we have shown that CitH3 is associated with relevant clinical outcomes in sepsis-ARDS in patients and in murine models of lung injury. CitH3 induces Caspase-1 dependent inflammasome activation in BMDMs and BMDCs *in vitro*, and leads to acute lung injury *in vivo*. Moreover, we demonstrated that treatment of septic mice with CitH3 monoclonal antibody significantly improves survival and sepsis-ALI in a murine model of CLP-induced septic shock, likely due to inhibition of CitH3 activated Caspase-1 dependent inflammasome pathway. Blockade of the CitH3-Caspase-1 pathway may represent a promising therapeutic target for septic shock and sepsis-induced ALI.

## Data Availability Statement

The raw data supporting the conclusions of this article will be made available by the authors, without undue reservation.

## Ethics Statement

The studies involving human participants were reviewed and approved by University of Michigan Institutional Review Board. The patients/participants provided their written informed consent to participate in this study. The animal study was reviewed and approved by University of Michigan Animal Care and Use Committee.

## Author Contributions

YL, TS, HA, YT, and PL contributed to the concept and design of the study. KS and TS provided the clinical data and samples. YT, ZW, QD, and BP contributed to the performance of the assays. YT performed all the statistical analysis and wrote the manuscript. YL, TS, and KS contributed to the revision of manuscript. All authors contributed to the article and approved the submitted version.

## Funding

This work was funded by grants from the National Institute of Health R01 (R01HL155116) to YL and HA and the Joint-of-Institute (Grant# U068874) to YL.

## Conflict of Interest

The authors declare that the research was conducted in the absence of any commercial or financial relationships that could be construed as a potential conflict of interest.

## Publisher’s Note

All claims expressed in this article are solely those of the authors and do not necessarily represent those of their affiliated organizations, or those of the publisher, the editors and the reviewers. Any product that may be evaluated in this article, or claim that may be made by its manufacturer, is not guaranteed or endorsed by the publisher.
