# Supplementary material for: Citrullinated Histone H3 Mediates Sepsis-Induced Lung Injury Through Activating Caspase-1 Dependent Inflammasome Pathway
Source: Front Immunol. 2021 Dec 7;12:761345. doi: 10.3389/fimmu.2021.761345 (PMC8688857; doi:10.3389/fimmu.2021.761345)
Supplement: Supplementary file 1 [file DataSheet_1.pdf]

Supplementary Material

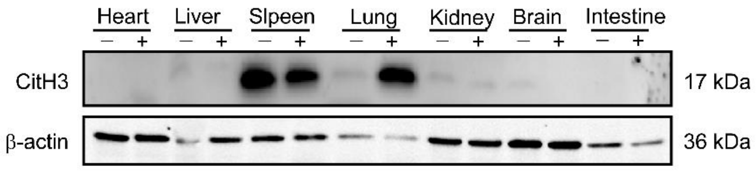

Supplementary Figure 1. Tail vein injection of CitH3 peptide induces most significant expression of endogenous CitH3 protein in lung tissue. WT mice were subjected to CitH3 peptide challenge, and euthanized at 24 h. Organs were harvested and homogenized for WB detection. 30 μg of protein lysate of each organ was separated on 10% sodium dodecyl-sulfate polyacrylamide gel electrophoresis. Representative Western blot images show the expression of CitH3 protein in heart, liver, spleen, lung, kidney, brain, and intestine.

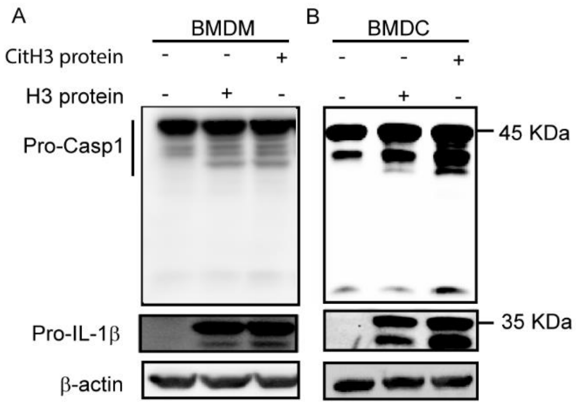

Supplementary Figure 2: CitH3 treatment induces significant elevation of pro-caspase-1 and pro-IL-1β. BMDMs and BMDCs were isolated from WT mice. Representative Western blot images of pro-Caspase-1 and pro-IL-1β in (A) BMDMs and (B) BMDCs after treatment with vehicle, H3 or CitH3 for 5 h.

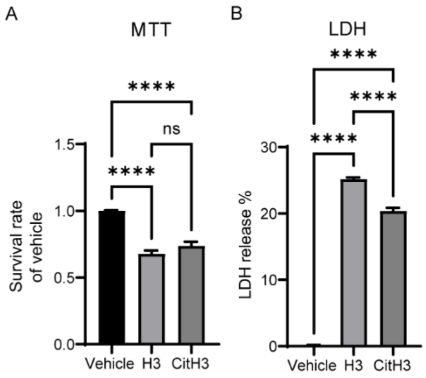

Supplemental Figure 3: CitH3 and H3 protein treatment induce BMDMs cell death. BMDMs were treated with vehicle, H3 or CitH3 protein for 5 h, the MTT assay (A) and LDH release in the supernatant (B) were measured.
